# Surgery

**Published:** 1825-07-01

**Authors:** 


### SURGERY.

1. *Wound of the Carotid Artery.** We shall give the particulars of this curious case, before we make any observations on the conclusions which are drawn or to be drawn from it.

*Case.* Charles A———, a sergeant in the army, aged 21 years, of thin form and lymphatic temperament, had experienced, almost from infancy, symptoms of hypertrophy of the heart—the pulsations of which were felt with great force, and to a great extent in the chest. On the 13th July, 1824, he received, in a duel, the thrust of a small sword immediately above the articulation of the right clavicle with the sternum, instantly followed by a profuse hæmorrhage of arterial blood, ending in syncope. The neck was muffled with handkerchiefs, and the patient was conveyed to the Hôpital St. Eloi. Half an hour afterwards, M. Delpech saw him. He had recovered from the syncope —vomited—and was pale and languid—pulse weak and irregular. The skin around the wound was puffed out by a mass of coagulated blood, and there was considerable intumescence of the front and right side of the neck. There was evident to the eye and to the finger a pulsation of the part. The situation and the direction of the wound—the form of the tumour, and other circumstances, pointed out the arteria innominata (le tronc innominé de l'artère carotide droite) as the probable seat of the injury. At all events, the wound of the vessel was below the level of the top of the sternum, in which situation there was no prospect of success from an attempt to pass a ligature round the vessel. The hæmorrhage was stopped for a time by the thrombus, and it was highly desirable to preserve things in their actual condition as long as possible. The coagulum which presented itself between the lips of the wound was not disturbed, and adhesive straps were passed around so as to support the edges of the wound, and prevent the escape of the coagulum, without, at the same time, making too great pressure. Two assistants were stationed with the patient to relieve each other, and make gentle pressure with their fingers on the site of the wound. The patient was

* Professor Delpech. Revue Med. December, 1824.

placed in bed, with his shoulders raised, and twenty ounces of blood were immediately taken from the arm. The whole of the neck was covered with a bag of ice, which was hourly renewed. Iced lemonade was ordered for drink. It was now nine o'clock in the evening, and the large bleeding had reduced the pulse to a very feeble state. Venesection was ordered to be repeated as often as the pulse rose. At one o'clock in the morning, great anxiety, oppression, pain at the top of the sternum and clavicles; inclination to cough, which was with difficulty resisted. The pulse had risen in force and frequency. *Twelve ounces of blood were abstracted.* At three o'clock, a renewal of the state of restlessness, and the pulse had regained strength. *Twelve ounces of blood taken.* At seven o'clock in the morning, he was visited by Delpech, who found the symptoms again indicating venesection, which was performed to *ten ounces.* At 11 in the forenoon, bled to *ten ounces.* At 1. P. M. *venesectio ad* ℥viij—*two grains of powder of digitalis every two hours.* 4. P. M. The arterial action had again increased, and the pulsating tumour had considerably enlarged, beating awfully. The clot was kept between the lips of the wound by main force. Pad and compresses applied to the mass of coagulum and over the wound, and secured by bandages, straps, and manual pressure. The digitalis to be taken every hour. *Bled, for the seventh time, to ten ounces.* At nine in the evening, (14th) agitation, increase of pain, rise of pulse. *Bled to eight ounces.* At one in the morning, (15th July) *bled to six ounces.* At 7 A. M. *bled to eight ounces.* *Three grains of digitalis every hour.* At 3 P. M. the pulse was only 50 in the minute, but getting full and sharp. The pain about the sternum, however, had increased, and the patient was bled, for the eleventh time, to *ten ounces.* This night was passed more calmly, and the patient had some sleep. 16th. At 7 in the morning, the pulse was 55 and feeble; but the action of the heart was energetic, and accompanied with pain throughout the whole of the *left* side of the chest. The ecchymosis had spread—the tumour had become harder and more coloured, with diffuse pulsation. Complains of cardialgia. *Bled to* 8 *ounces:*—digitalis reduced to two grains every hour. At 7 in the evening, although the pulse was at 50, it had become full, and *ten ounces of blood were taken.* This night was also quiet—the pains in the sternum diminished. 17th. At 7 in the morning the pulse was at 40, feeble, irregular, and occasionally intermitting. The ecchymosis extends—the breathing is freer—complains of cardialgia, particularly after taking the digitalis. At 2, P. M. the pulse has risen, though not more frequent. *Bled to* 8 *ounces.* 18th. At 7 in the morning, pulse 45—cardialgia and sickness. At 2. P. M. it was necessary to bleed again to 8 *ounces.* 19th. The pulse keeps low—some vomiting. 20th. The state of things was promising in all respects, but the patient rejects the digitalis with disgust. 21—to 24. Same state. Emollient glysters brought away great quantities of fæces. The aneurismal tumour gets harder and harder—the pulsations are obscure—the volume of the swelling rather diminished.—The ice applications are left off during the last four days. This day (24th) the patient complains of pain in the centre of the tumour. The coagulum is partly detached, and a sanguineous fluid oozes forth. *Bled to* 12 *ounces.* In the evening an accession of fever. 25th. The iced applications renewed—five grains of the sulphate of quinine to be taken twice in the forenoon. A greater discharge of sanguineous fluid to-day. In the evening another accession of fever, but weaker. 26th. Still an oozing of blood from the wound—but the tumour is less turgid. Three grains of quinine. No evening fever to-day. 27th—28th—29th, the oozing of decomposed blood continues, mixed with pus—pain throughout the tumour—and it is evident that suppurative inflammation has taken place all round *the coagulum*—The coagulum itself is virtually detached, and seems to supply the greater part of the sanguineous oozing. Some dangerous event was now dreaded. A great number of leeches were applied over the tumour, with the view of checking the progress of the inflammation going on within. 30th. At 3 P. M. hæmorrhage to about four ounces took place, in arterial jets. A new compress was applied, and the bleeding ceased externally, but continued to fill the cavity below, and painfully to distend the parietes of the tumour. At 7 in the evening, venesection to four ounces. The digitalis (2 grains) resumed every hour. In the night, a fresh hæmorrhage, which was again restrained by firm pressure. The volume of the tumour, however, is evidently augmented by each hæmorrhage. 31st. At 7 in the morning twenty-four leeches to the neck. 1*st. August.* The extravasation of blood in the cellular substance extends to the upper part of the sternum, and produces much pain. *Twenty leeches* gave relief. The oozing of bloody pus continues. 2d. Acute pains about the middle of the sternum, relieved by the application of 16 leeches. 5th. Violent palpitations of the heart—acceleration of the pulse. Digitalis in infusion. In the evening, relief—night tranquil. 7th. Diminution of the tumour—the patient feels very well, and craves for food. Two ounces of bouillie allowed. From the 8th till the 12th—the suppuration became more pure and copious, with progressive diminution of the tumour, in which, however, there was still a pulsation to be felt. The nutriment was allowed in larger quantity. From the 12th to the 24th—granulations shot forth in the place which the coagulum had occupied. The pulsation was only felt in the centre, and was very obscure at a little distance from thence. The suppuration is still copious. 26th. There was some fever to-day. The patient acknowledged he had procured some solid food privately, by which he was much incommoded. From the 26th of August to the 9th September, the patient was troubled with colics and diarrhœa. He was kept on a most rigid regimen, notwithstanding his remonstrances to the contrary. 10th September, every thing favourable. The tumour is reduced, to a very small size, and what remains is firm. There is still some pulsation about the site of the original wound. More nutriment was now allowed—the patient gained strength—left his bed—the wound healed—but a pulsation was still perceptible over the cicatrix, accompanied by a thrilling sensation, like that which is observed in cases of communication between an artery and vein. The patient left the hospital on the 3d of October, as well as could be expected after such a desperate injury. He was counselled to renounce the military profession—to avoid all strong exertions of mind, body, or voice—in short, every thing which might accelerate the circulation, or poduce fulness of the vascular system.

*Observations.* Professor Delpech thinks that the phenomena altogether, when taken in connexion with the site and direction of the wound, must leave no doubt that the carotid artery was wounded. This point, we apprehend, must be conceded to him. The case is an admirable illustration of what may be done by repeated bleedings, cold applications, and digitalis in large doses, for the repression of hæmorrhage from arteries of even very large calibre. We are very far, however, from thinking that the patient is free from all danger of aneurism. When he comes to have more blood in circulation than when he left the hospital, let him beware of a storm. Delpech himself indeed, does not seem quite easy as to the future. "Il est difficile," says he, "de dire ce que deviendront les choses dans l'avenir." Sincerely do we hope that the exertions which were meritoriously made to avert the immediate danger, may be successful in warding off any sinister consequences that might be reasonably dreaded in the sequel.

---

2. *Lithotomy.** Every practical suggestion from so expert an operator as Mr. Liston, will be attentively listened to by the surgeon. Mr. L. considers the lateral operation, when well performed, as the best possible mode of lithotomy. In this paper, our author proposes a method of proceeding "which undoubtedly obviates a great deal of danger." For the originality of the plan Mr. Liston does not vouch—but only for its disuse. Previously to stating this improvement Mr. L. dwells on the causes of death after lithotomy, which he avers to be rarely more than two—the exhaustion of the powers of life from the tediousness of the operation—and infiltration of urine into the cellular substance about the neck of the bladder, in the perineum and pelvis. Has M. L. never seen men die from hæmorrhage and its consequences? We have seen instances, where the effects of a severe hæmorrhage were never overcome by the constitution, and where the patients died at the end of three or four days, exhausted and exsanguined.

On the first class of causes (protracted operation) Mr. L. does not mean to comment. He observes, however, in another part of his paper, that even in the worst and most complicated cases of lithotomy, there can be no excuse for the patient being more than a very few minutes on the table. We have seen some of the most expert surgeons

---

* Remarks on the Operation of Lithotomy. By Robert Liston, Esq. *Ed. Journal, January,* 1825.

in Europe unable, with all their dexterity, to accomplish lithotomy under a quarter of an hour or twenty minutes. We certainly think that this *contest with time*, in surgical operations, is far beneath the ambition of a scientific surgeon. But to return. The extravasation of urine is the great danger, and Mr. Liston's improvement is for the purpose of obviating this. Mr. L. thinks that *inflammation* is often blamed, when *infiltration* is the cause of the bad symptoms. We do not see how infiltration can take place without inflammation following—at the same time, we grant that this last inflammation differs materially from common inflammation unconnected with infiltration. The causes which produce infiltration are various.—Thus, the external incision may be too high, so that there is no dependent passage for the escape of fluids—or it may be not in proportion with the internal incision; in consequence of which, extravasation takes place from the wound being blocked up by coagula, or its external lips glued together before the urine begins to flow. This last accident seems to be rendered more likely to happen by the old practice of securing the patient's thighs together, "the wound being previously covered up with dressings," as is often the case, according to Mr. Liston. We know not what is the practice, in this point, in Edinburgh; but we can aver that no surgeon of this metropolis thinks of applying any dressings to the wound, or of tying the thighs together.

Swelling about the wound will be apt to take place where there has been much difficulty in extracting the stone, and consequently where there has been some laceration and bruising of the parts. "If the inflammatory action has been general, after a short time the chance of infiltration occurring is very slight."

"In order to do away with this danger, and also to facilitate the extraction of the stone, the external incision should be made extensive. It should be made to pass well by the side of the anus. All stricture occasioned by the *levator ani* is to be relieved, the rectum being pressed to the one side by the finger of the left hand. The staff is then, and not till then, to be felt for in the upper part of the incision, under the symphysis pubis (where it is covered merely by the membranous part of the urethra), and the incision into the neck of the bladder made from thence. In making the incisions thus, the operator does not interfere with the management of the staff; the surgeon assisting keeps that instrument well lodged in the bladder, and hooked firmly against the symphysis pubis, so as to afford as much room as possible betwixt the urethra and rectum. It is not moved from *this position* from the time of its introduction, until the opening into the bladder is completed.

"The preliminary incisions are made, of course, with a sharp-pointed knife, and it is of little consequence whether those into the bladder are performed with a beaked one or not. The greater number of those surgeons who have studied this and other operations on the dead body are agreed, that the internal as well as external incisions can be very well executed with the common operating knife. An opening sufficient to admit the finger easily is made by simply pushing the knife along the groove of the staff, and raising it a little upon the point, and through this, a stone of a considerable size can readily be made to pass; if, however, the stone is found to be of a large size, a narrow probe-pointed knife will be found the most convenient instrument for enlarging the wound sufficiently." 29.

The causes of infiltration are numerous, but in order to obviate them effectually, our author recommends (and we entirely agree with him) the introduction of an elastic tube through the wound into the bladder. Mr. L. has been in the habit of using a piece of œsophagus tube, about 5½ inches long, and between two and three eighths of an inch in diameter, perforated by a common punch with a few holes at the end, which is to be inserted into the bladder. The tube should be introduced immediately after the operation, and retained by straps. It should be carefully cleared of coagula by a probe and lint, for the first few hours after the patient is put to bed, and should not be withdrawn till the track of the wound has had time to become consolidated—which is generally four days.

---

3. *Dissection Wounds.* Mr. Shaw has published a short paper on this subject, in the May Number of the Medical and Physical Journal, of which we shall here take some notice. He agrees with Dr. Duncan, that "the effects of wounds received during dissection, should be classed under two heads, forming cases which differ essentially from each other. The one seems to be of a specific nature, is attended with immediate danger, and is generally the consequence of examining a body a few hours after death; and, as far as my experience goes, most frequently follows dissection of the bodies of persons who have died with inflammation of some of the serous membranes. The other cases are more common, are less dangerous, and resemble, in many respects those with which all who have attended to hospital practice must be familiar:—I allude now to the consequences of wounds received in the dissecting-room during the examination of parts which are putrid."

Mr. Shaw thinks that means have now been discovered to *prevent* this last class of morbid affections, or at least to render them of comparatively little danger. This prevention is effected by means of injection of the bodies with solution of nitre and salt, previously to their being dissected. In consequence of this procedure, it is common for students to keep a body for eight or ten weeks, with scarcely any bad smell, the muscles of the limbs appearing fresh and florid. Previously to this plan being put in execution, frequent bad consequences resulted from dissection wounds—particularly in the spring, when the constitutions of the students had become deteriorated by a winter residence in town. "The only cases that have occurred during the last three or four years, proceeded from the dissection of the ligaments, after the limb had been kept for some time in water—or in preparing bones that have been macerated—being, in short, the only occasions on which the student is subjected to putrid matter not corrected by the solution of nitre and bay salt."

"The history of the effects of a wound made under these circumstances, is nearly the following:—The finger is scratched or pricked in the morning; there is not much pain at the time, but it gradually increases towards the evening; a little uneasiness is felt in the axilla, and next morning red lines can be perceived running up the arm. The finger is now excessively painful; there are often slight rigors, and general uneasiness; the countenance is anxious, and the tongue is sometimes furred, with head-ache; but still there is not much fever.

"The finger becomes rapidly swollen and livid, so as to call for immediate attention, and the general system is so much affected that we cannot be satisfied in considering the patient as suffering merely from a local complaint.

"I shall now offer a view of the general plan of treatment I have followed in such cases. The best local application is lint soaked in equal parts of laudanum and Goulard's lotion, which are to be used tepid at first. The lint should be applied in slips round the finger, and in larger pieces over the hand and up the arm; the whole of which should be kept on a pillow, the hand being on rather a higher level than the shoulder. Notwithstanding this soothing application, the pain sometimes continues so intense, that the patient begs to be allowed to try the effect of a poultice. If this be applied, a large quantity of laudanum should be put into it; but I have generally found that the patient, after a short trial of the poultice, gave it up for the wet dossils. The weight of the poultice seems to increase the irritation. In the course of the second or third day, the patient is so convinced that there is matter situated deeply, that he urges the necessity of cutting down upon it. This is a question of some difficulty. I have several times been disappointed in the expectation of finding matter, although I have cut down to the bone; but I believe it is safer to commit this error, than run the risk of allowing matter to lodge: it does occasionally form very rapidly; and if, under such circumstances, it be not evacuated, it keeps up a dreadful degree of irritation. It is well known that, when matter is pent up under the tendons, it quickly causes them to slough, and even the bone to exfoliate. I would, therefore, were there the slightest suspicion of matter being lodged, cut fairly down to the bone. From experience in my own person, I can state how great and how immediate the relief is from its evacuation, although the operation itself is most painful. By the fifth or sixth day, if the general treatment has been judicious, the bad local symptoms will probably have subsided; but the hand should, for three weeks at least after the severer symptoms have disappeared, be carefully nursed and kept in a sling, as a sort of erysipelatous inflammation is very apt to attack it, and to run from one finger to another, and to be increased by the slightest exertion. The swellings in the axilla must not be neglected; but they have never suppurated in any case that I have seen.

"The general treatment I have pursued, has been to keep the bowels in an active state, and this by a combination of calomel with the warmer purgatives, in preference to saline medicines; to keep the patient at first almost in a state of stupefaction, by laudanum and porter. This mode of treatment has been criticised; but I still recommend it, however it may be in opposition to the theories of the nature of this affection, because it appears to afford more relief than any other. While suffering severely under one of these attacks, I have, before going to bed, taken a large dose of opium, and nearly a pot of porter: in this way I have been relieved from the excessive pain, and have slept soundly, although, when I am in good health, a few drops of laudanum or a pint of porter would make me feverish and restless.

"I enjoin the patient to live well, and take more wine than he has been accustomed to; and, if the arm is not hurt by motion, to keep as much as possible in the open air. When the severity of the inflammation has subsided, I advise him either to go into the country, or to take occasional short rides out of town on the outside of a coach." 372.

We have already, in the review of Dr. Duncan's paper, expressed our opinion that the inflammation and pyrexia following poisoned wounds (for surely these deserve the name,) cannot safely be treated in the same depletive manner as common inflammations and their attendant states of fever. While bearing this general caution in mind, we should, however, be guided in each individual case by the phenomena presented in that case. When, for instance, the pain is highly acute, the parts swelled and inflamed—the pulse hard, full, and quick—the constitution vigorous, we surely *ought* not to proceed on the principle, of dosing the patient with laudanum and porter, in the same way as where the phenomena are of the low kind, and where the system appeared (as it often does,) labouring under a poison which it is striving to throw off. In these last cases, which are by far the most numerous, we should certainly prefer Mr. Shaw's plan of treatment, as it is most consonant with reason and with the character of the symptoms exhibited.

Our author never advises venesection in cases similar to those he has described above, and doubts the propriety of leeches, even when the inflammation extends up the arm. "He would trust principally to an assiduous use of the Goulard lotion and laudanum along the arm."

If the patient be in bad health, there will be danger of the local irritation increasing so as to cause foul abscesses. In such event he, Mr. Shaw, thinks the principles of treatment will be the same as in those cases familiarly known by the term of the "bad hospital hand"—in fact, nearly the same treatment as that which he has already described.

"The other class of cases is more serious, as it constitutes an affection to which the practitioner is more liable than the student, and one that is attended with immediate danger to life.

"All who have been in the habit of attending to morbid anatomy, must admit that there is more danger from a wound received during the examination of the body of a person who has died from any form of inflammation of the peritoneum, than of any other disease. So much have I been convinced of the truth of this, that I have for many years inculcated on the student the necessity of being particularly careful when examining a body, even though it is not putrid, where death has been caused by puerperal fever, the operation for hernia, or any form of peritonitis, or even of pleuritis.

"If, five or six hours after examining such a body, pain is felt in the finger, and a small pimple or a blush of red is found at the part, immediate attention should be paid to it. If the case proceeds in the usual manner, there will probably be a darting pain up the arm, which seems to fix more particularly in the shoulder or side of the chest. Within fourteen hours, the patient is very ill; he suffers a great deal of pain, and is anxious and alarmed. Red lines may generally be perceived running from the hand towards the axilla, but it sometimes happens that there are no marks on the arm, nor even on the finger. Indeed, the affection of the finger is occasionally so slight, that it is neglected, and the patient refers all his suffering to the shoulder and chest." 373.

Here Mr. Shaw relates the cases of Dr. Dewar and Mr. Hercey, published by Dr. Duncan, and already analysed in the 3d. number of this series.

In the discussions respecting these kinds of wounds, which have occupied most of the medical societies of the metropolis during the last winter, some anatomists and pathologists maintained that the bad consequences resulting from dissection wounds were totally unconnected with any poison introduced by the instrument, and entirely dependent on the irritation of the puncture in a deteriorated constitution. This appears to us to be a very lame doctrine. If the symptoms depended merely on the puncture, and the matters on the instrument had nothing to do with the said symptoms, how is it that we have not these effects constantly taking place in common life, among artificers, who are daily and hourly cutting themselves with various instruments, their constitutions too being very far from the best; whereas, the train of phenomena following dissection wounds is rarely, if ever seen among them? One anatomist, in a public society, asserted his opinion that no poison was introduced in dissection wounds, proving, as he imagined, this doctrine, by the fact that he had several times seen students cut themselves freely, while dissecting putrid bodies, without any bad effects resulting. This we conceive to be a very weak argument. For certainly the more free the cut, the less danger of absorption; and *vice versâ.*

The following are Mr. Shaw's therapeutical sentiments, in which, to a certain extent, we are inclined to agree:

"With regard to the treatment, the most important question seems to be, what is to be done during the first twenty-four or forty-eight hours? for, after this time, the peculiar character of the disease is in a great measure lost. The patient may fall into a low typhoid state, or he may labour under the symptoms of the erysipelas phlegmonoides; so that after a few days, there may be a question as to whether he is to be stimulated and supported, or to be reduced by bleeding.

"Are we to bleed in the first instance?—Is a man who is suffering under a poison to be bled, because he has a pulse at 140, rather hard and full, head-ache, anxiety and difficulty of breathing, and flushing of the face? Under almost any other circumstances, I believe no one would for a moment doubt the propriety of a copious bleeding, when such symptoms are present: but, may not the pulse be raised by a morbid irritation,—the anxiety and flushing of the face proceed from terror,—the head-ache from the same cause,—and the difficulty of respiration be owing partly to the same, and to the morbid condition of the principal respiratory muscles? But we seldom have the face flushed; it is generally pale and haggard, with a cold perspiration on the brow; and the tongue has not the white dry surface, nor has the pulse the hard stridulous beat, that marks the inordinate action which requires to be subdued by blood-letting. Proceeding on these views, and on the conclusions I have drawn from the accounts given of the effects of bleeding in the cases that are recorded, I have objected to venesection. I have also objected to local bleeding by leeches; for the swelling and inflammation of the arm is of a character that does not appear to be benefitted by such treatment; and although, from the cases recorded, we may conclude that local bleeding has been sometimes of advantage, still its good effects have not been sufficiently marked to induce me to run the risk of reducing in this way the vital energies of my patient." 377.

Mr. Shaw suggests the propriety of smearing the fingers with lard or pomatum, when examining the thorax or abdomen. He does not think that any one should be accused of effeminacy even were he to wear thin gloves during the examination of the body of a person who had died of peritonitis. "It is difficult to say what is the best thing to be done when the finger is wounded under these circumstances. Washing it well with soap and water, afterwards sucking it, and then bathing it in spirits or turpentine, would probably prevent any bad results."

"But, if this should be ineffectual, the moment the pain commenced, I would immerse my finger in equal parts of laudanum and Goulard's lotion; I would take a smart dose of calomel and antimony, and, two hours afterwards, a large dose of laudanum. If the pain still continued, I would bathe my whole hand and fore-arm with tepid laudanum and Goulard, take another opiate, perhaps with a little ammonia, and even force myself to swallow cordials in the form of warm negus, &c." 379.

---

4. *Dissection Wound.** The attention of the profession has been strongly drawn, of late, to this class of accidents. Cases and observations have been published through various periodical works, and the medical societies of this metropolis have discussed the subject, night

---

* Dr. Anthony Todd Thomson. Repos. April, 1825.

after night, with great interest, and with the good effect of eliciting a great number of curious cases and highly valuable remarks. We do not recollect that any medical man has published his own case, before this history which Mr. (now Dr.) Thomson has laid before his brethren. We shall, therefore, proceed at once to the particulars.

Early in the month of November of 1824, Dr. T. was requested to visit a lady who was so far gone in an inflammatory affection, that he hesitated to employ the lancet, "on which alone any prospect of benefit depended." She died on the following day. The inflammation was pleuritic. The lady was not pregnant. On examination, *post mortem*, the chest presented the usual appearance of destructive inflammation—namely, effusion and false membranes. In sewing up the body, Dr. Thomson received a slight scratch from a curved needle, on the first joint of the fore finger of the right hand. The wound was so slight that he paid no attention to it at the time. In the evening, however, or about 12 hours after the injury was received, Dr. T. observed some slight pain and inflammation in the part, which increased towards bed-time—awoke him from sleep—and kept him awake during the rest of the night, accompanied by a state of profuse perspiration. In the morning the finger was found to be considerably inflamed, with a small white spot in the centre of the scratch, from which a globule of pus was squeezed, after puncturing it with the point of a lancet. This gave much relief, and Dr. T. resumed his avocation. But, during his professional visits, he was attacked with rigors, loss of strength, and febrile symptoms. On returning home at two o'clock, he fainted, and afterwards soon became convinced of his alarming situation. He was sufficiently collected to perceive that prostration of strength was the prominent feature of the disease, and therefore wisely determined to rouse, if possible, the flagging energies of the nervous system. He ordered for himself a mixture of camphor, ammonia, and vinum colchici, of which he had taken two doses before his friend, Dr. Granville, called upon him. Dr. G. approved of the medicine, with the exception of the vinum colchici. The patient could not now convey a more accurate idea of his sensations than by comparing them to those which are said to result from the bite of the copra di capella, or other venemous serpent—or to those which he had once heard described by a person who had taken an overdose of prussic acid. His respiration was laborious, accompanied with acute pain under the ensiform cartilage, extending to a short distance along the sternum. The pulse was quick, vacillating, and struggling. Three grains of camphor and four of cayenne pepper were ordered by Dr. Granville, with hot water to the extremities, which were cold, as was the entire surface of the body, the features being shrunk and cadaverous. The finger was poulticed. A repetition of the bolus, with the means abovementioned, produced reaction, and some sleep was obtained during the night. On the following day, the pulse was 130, and small—skin hot and dry—some delirium. The bowels were freely opened by a bolus and brisk purgative of jalap and cream of tartar. The pain of the finger extended—

poultice. Three grains of James's powder and four of extract of henbane. Slept better this night. By continuing the use of the pills and keeping up a free action of the bowels for the three following days, he was so much relieved as to be able to sit up and take nourishment on the fifth day. The pain of the finger, which had hitherto been but inconsiderable, now became excruciating, and almost insupportable. The seat of pain was not in the site of the wound, but about an inch above it. Mr. Brodie made an incision, and a very minute portion of pus was discharged. By continuing fomentations for some days, suppuration was established, and recovery was completed. The following corollaries, Dr. Thomson thinks, are deducible from the facts connected with the origin and progress of this case:—

"1st, It is evident that diseases of an inflammatory nature, affecting the serous membranes, even when *unconnected with the puerperal state*, generate a virus, which continues active for some time after the death of the individual; and is capable, when introduced into the living system by inoculation, of exciting a dangerous degree of irritative fever.

"2d, It is probable, however, that a certain predisposition of the body of the dissector may be necessary for the production of this effect.

"In my own case, my health was not in the best state at the time of my receiving the scratch, as I had been previously much harassed both in body and mind, by the extent and nature of my professional duties.

"3d, That the effect of the introduction of this virus into wounds, or chops, or abrasions of the cuticle of the hands in dissection, is local inflammation and the production of a similar virus in the part, which, being absorbed into the system, diminishes so greatly the nervous energy, as nearly to destroy the action of the heart; and, thence, to produce congestions in the vascular trunks highly detrimental to the powers of life, and which prove not unfrequently fatal.

"If these corollaries be correct, two questions of great importance present themselves for consideration:—1st, In what manner, independent of care in avoiding wounds, is the influence of this virus to be guarded against in dissection? 2d, When inoculation has taken place, what plan of treatment is likely to prove most beneficial in overcoming the disease which follows?

"In considering the first query, it is obvious, that no description of gloves, nor any coverings for the hands of a similar nature, can be employed by the dissector; but I am of opinion, that the hands, if chopped or abraded, might be protected by rubbing them over with oil. It is a well-known fact, that the oil coolies at Tunis are rendered insusceptible of the contagion of plague owing to their bodies being constantly covered with oil, which can be explained only on the principle of non-contact; and as oil is impermeable by watery fluids, the probability is, that it would secure the chopped or abraded hands of the anatomist, in morbid dissections, on the same principle. But it cannot secure him from being wounded either by the scalpel, the needle, or the edges of fractured or of carious bones, or from the consequent inoculation; and, thence, the necessity of the second query.

"The wounds which are received in morbid dissections are seldom deep, and most frequently do not penetrate through the cutis vera. When this is the case, perhaps the most certain mode of preventing the threatened evil would be, instantly, to cut the portion of wounded skin with a *clean* scalpel, and to encourage a flow of blood by bathing it with warm water. Sometimes, however, scratches are received unconsciously, owing to the attention of the dissector being deeply engaged in the investigation of his subject; and the first notice he receives of the injury is from the local inflammation of the wounded part. It is then too late to have recourse to excision: what mode of treatment, therefore, should be adopted? Were I to reason from my own case only, I might consider it sufficient to refer to the treatment which it details; but the Profession has been instructed, by experience, not to depend upon the result of single cases. If the view, however, which has been given of the nature of the attack be correct—if the first effect of the absorption of the virus be to diminish the nervous energy, and, by thus weakening the moving powers of the blood, to permit congestions in the trunks of the vascular system,—it is evident, that, unless the balance of the circulation be restored, the functions of life cannot be continued. Reaction may take place by the powers of the habit; or the congestion may be removed by bleeding; or it may be overcome by reaction induced by rousing the nervous system by artificial stimuli, as in the case before us. If we trust to the powers of the habit, it is impossible to calculate upon the consequences: the resulting fever may be sufficient to endanger life; or organic mischief may occur, which would ultimately destroy it; and it is unnecessary, therefore, to reason upon the probable effects of the vis medicatrix in cases of this description. It only remains, therefore, to estimate the comparative advantages of bleeding and of stimuli. The opening a vein in the arm will undoubtedly relieve congestions in the vascular trunks within the thorax and abdomen; but, if these congestions have arisen from the nervous energy being diminished, by the introduction into the habit of a virus operating as a powerful sedative, it may be doubtful whether, after they have been thus relieved, the balance of the circulation will be maintained. If the moving power of the vascular system depends on nervous energy, the mere unloading the great vascular trunks, in which the blood has accumulated from the action of the heart being unequal to propel it onwards, owing to a paralysis, as it were, of the nerves, is not likely to restore that power; and, therefore, we must conclude, that the lancet is less efficient than stimuli, which are calculated to rouse and maintain the nervous energy. The result of the practice which has been generally employed in these cases, might be brought forward to determine the question; but the records of it are too scanty to admit of a satisfactory inference being drawn from it. In my own case, in which there was evidently no injury either to a nerve or a vein, the beneficial up the arm, even to the axilla. An evaporating lotion, instead of the effects of the stimulating plan, in the first instance, followed by active purgation, was decisive; and my friend Dr. Granville, to whose skill, judgment, and kind attention, I attribute my recovery, has found it so in several cases which have since occurred, and come under his care. It is, at all events, worthy of being tried by the test of experience." 286.

We believe it will be found in this, as in other fevers, that our practice, in each individual case, must be directed by the age and constitution of the patient, and the symptoms which are actually present. In the state of the system, as described by Dr. Thomson, and the actual phenomena present when Dr. Granville visited him, no man in his senses would have thought of venesection. On the other hand, had symptoms of strong reaction obtained, the lancet could not have been dispensed with. But, as we hinted on a former occasion, it should ever be borne in mind that the inflammatory excitement kindled up by a dissection wound, is not to be treated in the same way of forcible depletion as when the disease is simply a phlogosis of an internal organ, as of the lungs or brain, for example. In the fever under consideration, there is evidently a morbid poison introduced into the system, and Nature will, in most instances, be called upon for her utmost powers to throw off the virus. Her resources should therefore be economised, and the system no farther lowered than is absolutely necessary to protect the internal organs from destructive excitement.

---

5. *On Acupuncture. By M. Pelletan, Jun.** M. Jul. Cloquet has directed his attention to this curious therapeutical agent of late, in the Hospital Saint Louis, where rheumatisms and other painful diseases are very much congregated. The results of this ingenious physician's experience are now laid before the profession by M. Pelletan, Jun. in the January number of our valued Parisian cotemporary, the Revue Medicale, and Archives Generales for February, 1825.

Into the historical part of the document we do not deem it necessary to enter, especially as the history of acupuncture is already before the English public in Mr. Churchill's little work on the subject. It is only within these ten or fifteen years that acupuncture has been tried in Europe to any extent. Beclard, in Paris, has made numerous experiments, as detailed in the article "Acupuncture," in the new Dictionaire des Sciences Medicales; but it appears, from the observations and experience of Cloquet, that, hitherto, the needle has not been allowed to remain long enough in the parts—as it is upon this last circumstance that the principal chance of success depends. It is curious that a fact many years ago recorded by Berlioz, now comes in to the support of Cloquet's practice, and ought to have led to it before. In the year 1811 he published the case of a woman long harrassed with a nervous remittent fever, and who was relieved by acupuncture at the epigastrium.

---

* Revue Med. Janvier, 1825. Archives, Fevrier.

One of the needles, when plunged in, could not be extracted, and remained there. "During the whole of the time that the needle remained in the epigastric region, the patient was completely relieved of the nervous symptoms by which she had previously been tormented." Whether Cloquet took this hint or not, we are uninformed; but M. Pelletan observes, that if the free end of the needle be held between the fingers of another person, when it is engulphed in living parts, a sense of numbness, and even of contraction, will be felt in the fingers and up the arm—in short, from a series of experiments made before numerous and competent witnesses, and with delicate galvanometers, it appears that the needle becomes the conductor of a stream of electricity—and hence, probably, its *modus operandi* as a therapeutic agent. For the details on this head, we must refer to the journal already quoted, while we confine ourselves, in this place, to some of the practical results.

"When acupuncture," says M. Pelletan, "is practised for the relief of pain, it is very rare that any appreciable effect is produced in less than five or six minutes. He has never seen pain entirely removed under 15 or 20 minutes—while, in some instances, it was necessary to keep the needles in for several hours."

When pain is removed by the introduction of a single needle, it generally returns in a day or two, though with less intensity, and is again quickly removed by a repetition of the acupuncture. When a single acupuncture fails to relieve pain, a number of operations almost invariably prove successful, especially when performed for some days in succession. The effects of acupuncture have appeared to our author to be the more apparent in proportion as the operation was performed near the trunks of nerves leading to the seat of pain. It is highly probable, M. Pelletan thinks, that no bad consequence whatever would result from a fine needle traversing the trunk of an artery, a nerve, or a vein—at the same time it is more prudent to avoid such accidents.

Our author has repeatedly seen neuralgic, rheumatismal, and other pains, completely removed by one, two, or three operations of acupuncture. M. Jules Cloquet has performed this operation on more than 300 patients at the Hospital St. Louis, and in not more than twenty cases has it failed to produce more or less beneficial effects. In a very few instances the pain was exasperated; but in no case has any bad consequences followed the introduction of needles, though some of them were of considerable size. M. Pelletan thinks himself authorised to aver, that acupuncture is a remedy without inconvenience, and almost without pain, which nearly always cures neuralgic and rheumatic pains—and relieves, at least for a time, those depending on organic diseases.

We shall now detail a few cases in illustration of the foregoing observations.

*Case.* 1. N. Chartier, 64 years of age, of strong constitution, had had several attacks of acute pain in the lower extremities, which had given way to vapour baths. For the last three days he had experienced severe pains on the anterior part of the left leg, and during the preceding night he had no sleep whatever, from their violence. He presented himself to M. Cloquet on the 1st December, 1824, being a labourer in the hospital. A needle was introduced, an inch in depth, into the seat of pain, without his experiencing more than a slight sensation from its introduction. In three minutes, the original pain began to change into a sense of numbness. In twelve minutes, the pain ceased entirely—the needle was withdrawn—and the patient returned to his work.

*Case* 2. Adolphus Lerbrouse, 22 years of age, strong, and of sanguineous temperament, experienced, on the 8th December, a sensation of extreme cold in the left cheek, while coming out of a warm bath. To this succeeded acute pains in the left side of the head, with redness and slight swelling of the cheek, as also of the eyelids, the motions of the eye being performed with pain. By the 13th, the pains had become intolerable, and the patient, attributing them to a diseased tooth, had one of the molares extracted, which was perfectly sound. 14th. The sufferings were still farther exasperated, and without any intervals of ease. The lower jaw could not be moved. The night was passed in tortures, and without a wink of sleep—next morning he presented himself at M. Cloquet's wards in the hospital. The last-mentioned physician, in presence of M. M. de Lens and Kergaradec, introduced a needle into the middle of the cheek, with its point directed towards the origin of the facial nerve. To the free end of the needle was fixed a metallic conductor, plunged into salt water. In 8 minutes, pressure could be borne without increase of pain—the motions of the jaw and of the eye became in some degree practicable—the redness of the parts diminished. In twenty minutes the needle was withdrawn—all pain had ceased—the patient only felt a slight sense of numbness in the jaw—could take food—and, in two days, the swelling itself was entirely dissipated.

*Case* 3. Peter Colas, aged 42 years, a labourer, of strong constitution, had been subject to pain in the right knee for 5 years past. During the last three weeks the pain had increased in violence and in extent, spreading successively to the lumbar region and to the external part of the thigh and leg. The patient was forced to give up work, and could only hobble along by means of a stick. Such was his condition when he presented himself at the hospital, on the 28th December. Professor Cloquet, in the presence of several eminent physicians and surgeons, introduced a needle into the lumbar region, and another between the tuber ischii and the great trochanter. In ten minutes, considerable ease was procured—and, in a few minutes more, the pain of the leg entirely disappeared—the motion of flexion and extension became facile. In an hour the patient could walk with ease, the needles still in—and, the lumbar pain having subsided, both needles were withdrawn—the patient felt himself perfectly well—and walked home without any impediment. On the 30th, the pain returned as acute as ever, but only in the leg. A needle was introduced into the part affected—In ten minutes the pain was mitigated—and in a quarter of an hour it had entirely ceased. The patient went home, and did not afterwards apply.

*Case* 4. Stephen Delauny, 38 years of age, of large stature and robust constitution, had strained his loins three months previously in lifting a heavy weight, which strain was succeeded by some pain in the part. This pain afterwards shifted to the leg, where it was very violent, and extended down to the foot, accompanied by a sense of numbness and stinging. These came in paroxysms, and forced him to abandon his employment. He had passed two or three sleepless nights previous to his presenting himself at the hospital on the 10th December, 1824. M. Cloquet introduced a needle, with a conductor, into the left leg. In five minutes the pain was increased, so as to make the patient cry out. In twenty minutes a sense of heat and constriction was felt in the limb, with some diminution of the pain, which was still more severe than before the introduction of the needle. In three quarters of an hour, there was a notable relief. At the expiration of an hour the patient was ordered to walk; but this brought back the pain as violent as ever, especially in the sole of the foot. In this place another needle, with a conductor, was introduced. In an hour and a half from the beginning of the operation, the needles were withdrawn—the patient was able to walk better than before—but still felt darting pains, from time to time.

13th. The patient returned this day, saying that he had been better, but the pain was now chiefly felt at the top of the fibula. A needle was introduced at this part. Little impression was made on the pain by this introduction, though it was kept in for half an hour. On the 17th and 20th fresh introductions of the needle—the last one being so successful that the patient stamped his foot on the ground without feeling pain. On the 23d, the patient having resumed his occupations, and having experienced great fatigue, some heat and pain returned in the leg, but were dissipated by one more introduction of the needle.

The foregoing facts must be considered as authentic, being not only published by honorable men, but witnessed in a public hospital by numerous spectators. It is not impossible that there may be something in acupuncture—for assuredly—

——— "There are more things in Heaven and Earth
"Than are dreamt of in our philosophy."

P. S. Since the above was translated and condensed, we have met with another hospital report on the same subject, from the practice of M. Bally, in La Pitié, by Dr. Meyranse. Of this report it will be necessary to take some notice.

It appears that the needle made use of at La Pitié is constructed of steel, but rendered ductile by heating it in the flame of a candle, and then suffering it to cool slowly. Care is taken to avoid the trunks of vessels or nerves—nor is it ever attempted to introduce the needle into any of the viscera, as has been ridiculously and falsely reported in this country.* The experiment, indeed, was tried on animals, but the results were not such as to countenance such a hazardous proceeding in the human subject.

The first trials of acupuncture, at La Pitié, were in cases of chronic rheumatism, where the pains were not of a very severe kind, and where the swelling, when it existed, was rather of the œdematous than of the common inflammatory character. The patients were free from pyrexia, and there was no disturbance of any internal function. In contrary circumstances—that is, where the complaint was not confined to one or two joints, but was, on the other hand, to be seen in several parts of the body, with marks of constitutional excitement, the acupuncture was found to be unavailing. We shall now proceed to notice some cases.

*Case* 1. A man, aged 45, had a swelling of the right knee, with a sense of numbness, and difficulty of moving the joint. There were no constitutional symptoms. Various means had been tried to dissipate the local complaint, but without effect. Acupuncture was practised three times, with four needles each time, by which the numbness and stiffness were entirely removed—the swelling remaining the same.

*Case* 2. Conchoir, of irritable constitution, was subject to rheumatism in cold weather. He came into hospital for a gastric affection, and on its decline, became affected with rheumatism in the deltoid muscle of the left arm, and in the wrist of the same side. These pains were ushered in by some constitutional symptoms, as cold chills, reaction, &c. The pains continuing unchanged during three days, M. Bally proposed acupuncture, and three needles were introduced into the parts affected. In one hour afterwards, to their astonishment, the pain had entirely ceased—the motion of the wrist was rendered more free—and the following day the patient was so well as to consider himself cured. In fact, the pain returned no more, and the swelling had entirely disappeared by the sixth day from the acupuncturation.

*Case* 3. A man, 43 years of age, was troubled with a rheumatic affection of the lateral and posterior part of the neck, the pain being increased by pressure, motion, friction, and even changes in the weather. It was also worse in the night than in the day. It was accompanied by a sense of coldness and numbness of the part. There was no pyrexia. Acupuncture, with four needles, was practised three days in succession, and complete success was the result.

*Case* 4. A man, aged 55 years, was affected for three months with

---

* In one case a needle was supposed to have penetrated the lungs, if not the pericardium, but not designedly. There was a considerable degree of motion communicated to the needle corresponding with the cardiac pulsations. The needle was withdrawn, and no ill effects followed. *Ed.*

severe rheumatism, the pains being acute, lancinating, and often causing the patient to cry out. His limbs were more or less swelled—the skin dry, hot, especially the parts affected—there was thirst, head-ache. The rheumatic pains were exceedingly wandering; but during the last month their principal seat was in the loins, where they rendered stooping or other motion of the spine impossible. Four needles were plunged into the mass of muscles on each side of the spine, to the depth of two inches. Two hours having elapsed, the patient observed that the pain had almost entirely gone. In the night there was no exacerbation of the complaint. Next day no pain was complained of, and the man could bend his spine, with only a slight degree of uneasiness. Four more needles were introduced, and this operation completed the cure.

*Case* 5. Lenoir, aged 35, caught cold, while working in the King's garden, and was seized with rheumatic pains, of an acute character, the joints being burning hot, and pyrexia being present. It was wished to try the effects of acupuncture under these circumstances. Eight needles were therefore implanted in different inflamed joints, as the wrist, elbow, and knee. Neither the local nor the general symptoms were, in the least, influenced by the operation. The pains and the pyrexia continued the same. The operation was performed again, the next day, but with similar want of effect.

So much for rheumatism. Some cases are next related, where acupuncture was applied for the relief of neuralgic affections, which differ considerably in nature from those just stated.

*Case* 6. A countryman, 45 years of age, was subject for a long time to neuralgia. During the last fortnight he experienced acute pain in the anterior crural nerve, extending from the upper part of the thigh down to the ancle. Various means had been tried without relief. Four needles were introduced at the upper part of the thigh, near where the anterior crural nerve emerges. During the operation the patient experienced a sense of coldness and numbness of the inner part of the thigh and leg. The pain diminished sensibly under the first acupuncturation. A second operation was performed, and the pain entirely ceased. The patient, however, was kept in hospital eight days more, but no return of the neuralgia occurred.

*Case* 7. A young man had suffered from severe pain about the sacro-sciatic notch, spreading occasionally to the sacrum, and to the other parts of the hip. Latterly it had propagated itself downwards, in the direction of the fibula, towards the foot. Various remedies had been tried in vain, or only with momentary relief. Acupuncturation was performed in the region of the glutei muscles, and afforded, the first time, considerable solace. The needles were left in the flesh for an hour and a half. Three days afterwards the operation was repeated, and the patient was completely cured of a neuralgia under which he had long suffered.

*Case* 8. A man, named Paché, had laboured for the space of two years, under a neuralgic affection of the chest, the precise limits or site of which it is very difficult to define or describe. When touched ever so lightly on any part of the thorax, he experienced acute pain, and nervous excitement. The pulse was always extremely hard, so as to resemble the vibration of a wire. Yet the appetite was good. Every means were tried to relieve this unpleasant state, but all without effect, when M. Bally determined to try acupuncturation. This operation was three times performed, but without either aggravation or diminution of the complaint.

*Case* 9. A young man, 24 years of age, had suffered, during three months, from a neuralgic affection of the plantar nerves of the right foot. At times the pains were excessively acute, especially in the night. When he entered the hospital the sufferings were peculiarly severe. Three needles were implanted into the part affected, and produced decided relief—so much so, that the patient was quite astonished at the change. The sequel of the case, however, is not detailed.

*Case* 10. A man, 64 years of age, was affected with neuralgia of the orbito-frontal branch of the fifth pair. The pain would commence at the supra-orbital foramen, and spread like a fan over the forehead, never being entirely absent, but sometimes being more acute than at others. This state had continued 20 days. Leeches, opiate embrocations, &c. produced no good effect. Two needles were inserted in each temple, and allowed to remain half an hour. They gave temporary relief; but in two hours the neuralgia returned as violent as ever. Next day four needles were introduced, and kept in an hour. There was sensible diminution of pain; but it returned in three hours. The third day the operation was repeated, without producing even a momentary mitigation of the man's sufferings. Acupuncture was not therefore repeated.

This operation was tried in several cases of paralysis, especially hemiplegia, but without affording any benefit—as, indeed, might have been expected. More success was obtained from this measure in cases of cephalalgia and hemicrania.

*Case* 11. A man of bilious temperament, experienced excruciating hemicrania, which he compared to the driving of a sharp instrument into the skull. The pain, without being periodical, yet varied in intensity. The paroxysms generally continued three or four hours. Needles were introduced at the commencement of an attack, and mitigated much the pain. The operation was therefore repeated three days in succession. At the end of this time the hemicrania was cured, and it did not again return. He had been affected with the complaint three months.

*Case* 12. A man had been long subject to severe labour, and had latterly experienced much chagrin. He was attacked frequently with acute pain in the right half of his head. Acupuncture was practised on a day when the complaint was extremely severe. It was remarked that the temporal artery throbbed violently on the affected side. Two needles were introduced into the temple of that side. The hemicrania ceased, and never afterwards returned.

Some cases have lately been treated by acupuncture in this country, and with success. We do not therefore join in the cry of ridicule which some people have raised against the remedy. If we proscribe every remedy, the operation of which we do not comprehend, we shall have but a small list to work with!

---

6. *Extirpation of the Uterus.* In the April number of our respected northern cotemporary, two cases of this horrible operation are related from the German. The results are not calculated to encourage future attempts of this kind—at least under similar circumstances. The removal of a *prolapsed* uterus is quite a different thing, and has, we all know, been successfully performed. But the extirpation of an open cancerous womb is, what we prognosticate to be, a very unfeasible operation. Do we see an open cancer of the mamma removed with success?—Very rarely. And surely it must be still more improbable that an ulcerated uterus can, with prospect of recovery, be cut out. Here follow the cases, however, and our surgical brethren must judge for themselves.

*Case* 1. *By Dr. Siebold of Berlin.* Caroline Louisa ———, 38 years of age, married, and had been twice pregnant—once she miscarried, and once she went her full time—laboured under a discharge of blood from the uterus for the last two years, with frequent pains deep in the left side of the pelvis. For the last six months she was confined to bed, there being a constant discharge of black-coloured blood from the uterus, with constant dull gnawing pain in the pelvis and thighs. She was much emaciated—the face fallen in and tinted yellow—the eyes sunk—the pulse small—palpitations of the heart—swelling and hardness of the inguinal glands—right hypochondriac region hard, swollen, and painful on pressure. On examination, the uterus was found to be of a strong hardness—the vaginal portion in part destroyed, and the remainder torn and wrinkled. Each examination was followed by long-continued hæmorrhage and palpitation of the heart. Such was the patient's condition in the middle of April, 1815. Examinations after this could only be made *per anum*, and they shewed pronatio in conjunction with retroversio uteri, both body and fundus of the organ being in a state of scirrhous induration. She submitted to the operation on the 19th April.

*Operation.* The patient was placed nearly as for the operation of Lithotomy. A female catheter was introduced into the bladder and held by an assistant, who also pushed up the intestines as much as possible.

"The operator was seated in front of the patient, and having felt the catheter through the forepart of the vagina, he determined on making the first incision on the right side. For this purpose he introduced two fingers of the left hand into the vagina, immediately behind the arch of the pubes, and directly on the vaginal portion of the uterus, and along these he carried the instrument, with which he made an opening in the vagina, the catheter at this time being held as in the operation for stone, a little to the left side. The incision was now widened, so that the fingers could be got behind the womb, and the vagina separated in this part from the vaginal portion of the uterus. At this stage of the operation a very peculiar sound was heard, something similar to what is heard on cutting through hard cartilage. The operator now took the knife out, and introduced along his fingers a small polypus-scissars, with which he separated on this side the uterus, from the body quite back to the fundus, from its lateral connexions. The intestines could now be distinctly felt, but they did not in the least protrude. The patient was refreshed with some wine and water, and then the operation on the left side was commenced. Two fingers of the right hand were introduced into the vagina, and along these Savigny's knife was carried, with which the separation of the vagina, from the uterus close behind the pubes, was begun, the assistant now holding the catheter to the right side. But on reaching here, the posterior part, where the retroverted fundus of the uterus was situated, there was such a scirrhous mass, that the separation with the knife would not succeed. It occurred to Dr. Siebold then, that Osiander, in his operation, had used an instrument resembling a sharp chissel *(scharf Meissel)*, which he tried, and, contrary to his expectation, separated the uterus from the surrounding parts. The operator then attempted to draw the uterus towards him, but it slipped out of his hand, and ascended so far, that he could scarcely touch it with the point of his finger. Through the rectum, however, it could be distinctly felt, and therefore Dr. Siebold directed an assistant to carry his fingers up the gut and fix the uterus, whilst he pressed the uterus down from outwards, and at the same time endeavoured to lay hold of it with a pair of polypus-scissars; but all his attempts were fruitless. He now saw that the uterus was only to be reached by the introduction of the whole hand, but the vagina was so narrow and contracted, as not to admit of this. The operator, therefore, immediately divided from left to right, with a scalpel, the perineum, so as to be able to introduce his hand, which enabled him to reach the fundus of the uterus; he then separated all the attachments on the left side, and the uterus fell into his hand. The quantity of blood lost was about six ounces. On the removal of the uterus, and as soon as it was ascertained that the intestines did not protrude, a piece of lint wetted with vinegar was inserted into the vagina. After a short interval, and again ascertaining that the intestines were in their natural position, some lint dipped in a solution of alum, together with some moistened with oil, was introduced into the vagina; over these a sponge and compress, and lastly a T bandage. The patient was conveyed to a neighbouring bed, and placed in the horizontal position, with the shoulders somewhat lower than the rest of the body. The operation lasted twenty minutes; and during the whole time, the patient's courage did not forsake her. An anodyne draught was administered, and the strictest rest enjoined." 400.

Several times in the evening the patient was attacked with vomiting. She slept but little—and next morning the pain in the lower part of the abdomen was very acute and constant, with quick pulse, and countenance covered with cold clammy sweats. She dragged out a wretched existence sixty-five hours from the operation, and then expired.

*Dissection.* The small intestines were found inflamed, with effusion, such as is seen after puerperal fever, throughout the abdomen. The bladder and rectum, though not injured during the operation, were inflamed. The mesenteric glands were indurated and swollen. The right lobe of the liver was full of hydatids. The extirpated uterus was a mass of disease—the body and fundus of strong hardness—the vaginal portion destroyed by cancerous ulceration.

*Remarks.* We quite agree with our northern cotemporary that, in this operation, "there was no chance of success, nor was it to be expected that the patient would derive any benefit." In short, a more cruel, bloody, and ill-judged operation is not, we think, recorded in the annals of surgery. The state of the right hypochondrium shewed that organic disease existed there independently of the uterine cancer—therefore there could be no chance of success.

*Case* 2. This case was under much more favourable circumstances, but not more fortunate in the result. The lady had borne several children, and had, for many months, exhibited symptoms of organic disease of the womb. It was on the 9th of January, 1824, that Dr. Holscher was called to her. He found the countenance pale, but not of that yellowish earthy tint so common in carcinoma uteri. Her appetite was good, but she had much thirst. The abdomen was soft and free from pain on pressure, except over the uterus. On examination, he found the uterus encircled with projecting fungi. The external parts of generation were free from disease, and on examination, *per anum*, it was found that the surrounding parts were sound also. The patient was sensible of the nature of her complaint, and willing to undergo any operation that might be proposed.

Dr. Holscher considered that the removal of the scirrhous parts of the uterus alone afforded but a precarious prospect of cure, and therefore determined on a total extirpation.

*Operation.* Dr. H. conveyed a scalpel along the middle and fore finger of the left hand to the os uteri. The instrument was then carried round the fungi, and the uterus separated from nearly the whole of its connexion with the vagina. Five or six ounces of blood were lost in this operation. With the left hand he now seized the carcinomatous mass, but was unable to pull it down. He next tried to pull down the uterus by means of hooks, but in vain. He then determined to remove the carcinomatous mass out of the way. After the extirpation of this mass, a slight hæmorrhage took place, which was easily stopped. The uterus was now more easily got at, and the operator introduced a sharp-pointed knife as far back as the fundus. "An opening being made, the circular knife was introduced, and the uterus separated from all its lateral connexions, after which it was easily removed." The operation lasted 35 minutes, and only nine or ten ounces of blood were lost. The intestines did not protrude. The patient was much enfeebled (and no wonder, Heaven knows!) by the operation. She passed a restless night, and next morning was attacked with vomiting. The abdomen became distended—pulse feeble—and in 24 hours death relieved her from her sufferings. On examining the body, it is said that the *opening* in the peritoneum had closed to the size of a hen's egg—and nothing wrong was seen in any part of the pelvis.

Our cotemporary of the north is "almost inclined to think, with Dr. Holscher, that had the operation been performed a month earlier, it would have been attended with a different result." We do not think so. We consider the extirpation of a uterus not previously protruded or inverted, one of the most cruel and unfeasible operations that ever was projected or executed by the head or hand of man. We have recorded these cases, rather as beacons to guard against attempting things which may reasonably be considered as beyond the boundary of man's power, than as incentives to operations that are calculated to substantiate the charge of cruelty, too often, and perhaps not always without reason, brought against the surgical practitioner.—We are very far from discouraging bold or untried operations; but there is a line, beyond which it may not be prudent to go, even should a solitary instance or two of success rise up as precedents to bear out the operator. The human mind, in the great mass, is weak and easily prejudiced. Operations of a terrible kind, and of a revolting nature, such as that of *chisseling* out a diseased uterus agglutinated to the neighbouring parts, are not ill calculated to excite in the public mind, and in private individuals, an aversion and terror towards operations of infinitely less danger, and where success might be extremely probable. In this way, let it be recollected, our temerity may cause the death of many, by the hopeless attempt to save a few.

---

### 7. *Partial Amputation of the Hand; with Reflections on Intermitting Hæmorrhages after Operations.* By M. Benaben.

*Case.* Trinqué (B.) a farmer, aged 22 years, of very sanguineous temperament, had his left hand severely lacerated by the bursting of a musket, on the 21st December. A surgeon was called in, who merely removed some pieces of metal, cleaned, and dressed the hand. Hæmorrhage took place in the night, and M. Benaben was summoned. He suppressed the bleeding by pressure on the radial artery. Next day he examined the hand, and he found the metacarpal bones supporting the thumb and forefinger totally shattered, and that of the middle finger greatly injured in its upper extremity where it joins the carpal bones. He therefore determined to save some of the fingers if possible. Amputation was performed by passing a bistoury between the radius and os naviculare, cutting the tendons, ligaments, and radial artery—passing the knife between the os naviculare and os lunare, and along the edge of os magnum, so as to disarticulate the three first metacarpal bones. The two first were entirely removed, with the corresponding fragments of fingers—the superior half of the third was also removed, leaving a portion to sustain the middle finger, which it was hoped might be preserved. An irregular wound was the consequence of these sections. The end of the radius was covered as well as possible, and the parts dressed. The patient, who was till now pale, recruited, and even became heated. Some blood was taken from the arm, and repeated on the 23d, the day after the operation. On the 25th, the dressings were removed, and the wound looked satisfactory. There was considerable febrile excitement. The bleeding was repeated, and aperients given. 26th. Again bled, there being much fever, with tumefaction of the parts. 27th. Venesection. 28th. The inflammatory symptoms are yielding. Another small bleeding. In the evening there was a determination of blood to the head. 29th. Epistaxis. The wound looks well, the suppuration healthy. 30th. The parts are united over the extremity of the radius. January 1. In the evening there was some fever, followed by an exhalation of blood from the unhealed surface of the wound. 2d. There was some pain in the parts—in the evening considerable febrile heat, with thirst, and quick pulse. At the same hour as on the preceding evening there was a more abundant discharge of blood from the wound. The 3d. of January passed without event; but in the night another hæmorrhage took place. The ligature of the radial artery came away on the 4th. From this time all went on well, and the patient was discharged on the 28th February. The little and ring fingers recovered their powers, and the middle finger had not entirely lost its function. The patient was therefore able to work at his usual avocations.

M. Benaben's reflexions on the periodicity of the hæmorrhage, contained nothing worthy of extraction in this place. It is evident there was a periodical febrile movement in the system, relieved naturally by the hæmorrhage, as the former inflammatory systems were kept under by the repeated bleedings. The family surgeon had determined on amputating the arm above the wrist, before M. Benaben's arrival. The plan of the latter surgeon was evidently the best, as the event proved. Two or three fingers are better than none.—*Revue Med. Mars.* 1825.

8. *New method of treating Compound Fractures.** Every one, says Baron Larrey, knows the advantages which result from *infrequency* of dressing wounds, especially where the first dressings are judiciously applied. Many a time has he seen wounded soldiers carried some hundreds of miles, after battle, without having their wounds dressed—and when arrived at their destination presenting the said wounds nearly cicatrized, or in the fairest way for being so. These facts led our author to employ somewhat similar procedure, not only in simple, but also in compound fractures. This plan consists in leaving on the first dressings throughout the whole course of the cure. The bad effects, he observes, of daily dressing the wounds in compound fractures, are exceedingly great. These dressings disturb the limb, and increase the inflammation of bones, ligaments, and muscles, often causing high traumatic fever or abscesses, in some of the internal viscera, as the liver and lungs. The Baron's indications then are; *first* to scarify, or more properly speaking to *disembarrass* all such wounds, if contused, of foreign bodies, broken bones, &c. incising those parts that are put too much on the stretch—cleansing out all extravasated blood—securing arteries—washing the wounds, and leaving the parts as clean as possible. The limb should then be laid on a part of the apparatus properly prepared, and the fractured bones nicely adjusted. The edges of the wounds are next to be brought as much together as possible, by compresses with holes in them (au moyen de linges fenetrés,) and spread with ointment, over which lint is to be laid, and then more compresses soaked in any styptic glutinous liquor. These dressings should be so adjusted as to fit with great exactness to every part of the limb's periphery. They supply the place of splints. When the compresses are all on, then the dressings should be finished by an eighteen-tail bandage. These dressings should be allowed to remain on during the whole period of the cure. The Baron asserts that, with proper constitutional treatment, the inflammation will be very trifling, and hardly ever such as to require the slackening of the bandages. If this be the case—if no swelling, tension, and inflammation result from this mode of managing fractures, we have no doubt of its utility. But we can hardly persuade ourselves that so little swelling can follow any mode of dressing, so closely adjusted to the limb as the above, as not to require a change when the inflammation comes on. This, however, is a point which is easily ascertained in practice. Two cases in illustration are related by the Baron; but they need not be detailed here.

* M. Le Baron Larrey. Journal Complement. January 1825.

